# The Profile of Glucose Lowering Therapy in Persons with Type 2 Diabetes Mellitus in an Aging Russian Population

**DOI:** 10.3390/jpm12101689

**Published:** 2022-10-10

**Authors:** Sofia Malyutina, Elena Mazurenko, Ekaterina Mazdorova, Marina Shapkina, Ekaterina Avdeeva, Svetlana Mustafina, Galina Simonova, Andrey Ryabikov

**Affiliations:** Research Institute of Internal and Preventive Medicine—Branch of the Institute of Cytology and Genetics, Siberian Branch of Russian Academy of Sciences, 630089 Novosibirsk, Russia

**Keywords:** diabetes mellitus type 2, glucose lowering therapy, glycemic control, HAPIEE cohort, population

## Abstract

We aimed to analyze the profile of glucose lowering therapy (GLT) in persons with diabetes mellitus type 2 (DM2) in an aging Russian population. A random population sample (*n* = 3898, men/women, 55–84) was examined in Novosibirsk, during 2015–2018 (HAPIEE Project). The design of the present work is a cross-sectional study. DM2 was defined in those with a history of DM2 receiving GLT, or at a level of fasting plasma glucose (FPG) ≥7.0 mmol/L. The entire DM2 group was included in the analysis (*n* = 803); of these, 476 persons were taking GLT and were included in the analysis at stage 2. Regular GLT medication intake for 12 months was coded with ATC. In studied sample, the prevalence of DM2 was 20.8%. Among subjects with DM2, 59% of individuals received GLT, 32% did not. Glycemic control (FPG < 7.0 mmol/L) was achieved in every fifth participant with DM2 (35% in those receiving GLT). In frequency of GLT use, biguanides ranked in first place (75%), sulfonylurea derivatives in second (35%), insulins in third (12%), and iDPP-4 in fourth (5%). Among those receiving GLT, 24% used combined oral therapy, and 6% used insulin-combined therapy. In conclusion, in a population sample aged 55–84 examined in 2015–2018, glycemic control was achieved in every fifth participant with DM2, and in every third participant receiving GLT. The proportion of participants using new GLT drugs was small, and there was a lack of HbA1c monitoring for intensive glycemic control.

## 1. Introduction

Diabetes mellitus (DM) is a global problem, due to an annual increase in its prevalence in the world. According to the latest data, the global number of patients with type 2 diabetes (DM2) has reached 463 million people [1]. In the Russian Federation (RF), a similar situation has been observed, and, according to the Russian Diabetes Register, this figure had reached 4.58 million people (3.1% of the population) by 1 January 2019 [2]. According to our early data, DM2 was found in 11.4% of subjects in a population sample aged 45–69 years in Novosibirsk [3,4].

The financial burden for people with diabetes and for society as a whole is growing due to lifelong daily care, glycemic control, treatment of diabetes complications and hospitalizations, as well as by indirect costs associated with reduced quality of life and disability.

The most dangerous consequences of DM2 include vascular complications—nephropathy, retinopathy, lesion of coronary, cerebral and lower extremities arteries. These complications are the main cause of disability and mortality in patients with DM2. Among the methods of evidence-based medicine that have demonstrated the highest effectiveness in reducing the risk of diabetic complications, the achievement of targeted glycemic control is the most essential [5].

Early achievement of stable glycemic control is a key component of effective management of patients with DM2 [6,7,8]. A prospective study, UKPDS, demonstrated that absolute reduction in glycated hemoglobin (HbA1c) by 1.0% was associated with a 21%, 14% and 37% reduction in the risk of diabetes-related death, myocardial infarction and microvascular complications, respectively [6].

Based on these data, most clinical guidelines recommend a target level of HbA1c <7.0% or ≤6.5%, depending on additional factors such as age, duration of diabetes, comorbidities, and risk of hypoglycemia [9,10,11]. When a patient’s HbA1c level is above the target level for more than 6 months following the last update of therapy, an intensification of treatment is recommended [11,12]. Despite established guidelines and the availability of modern glucose-lowering medications, there is evidence of poor achievement of glycemic targets and untimely intensification of therapy [13,14].

The achievement of target levels of HbA1c was assessed in the EUROASPIRE I–V [15] and NHANES [16] studies. According to the summarized data, about 50% of patients with DM2 did not reach the target levels of HbA1c. The 3-year project DISCOVER, which studied 15,992 subjects aged >18 years with DM2 who received standard medical care as determined by their treating physicians in 38 countries including Russia, confirmed this fact [17]. In particular, stable high levels of HbA1c were observed in patients with DM2 at the beginning of their second-line therapy: about one half of patients had HbA1c levels >8.0% and more than a quarter had HbA1c levels >9.0%. Overall, <20% of patients had an HbA1c < 7.0% [17]. In the NHANES study, the percentage of subjects with diabetes who achieved glycemic control (HbA1c < 7%) decreased from 57.4% (in the period 2007–2010) to 50.5% (in the period 2015–2018) [18]. According to the Russian Diabetes Register in 2017, the distribution of DM2 patients by HbA1c level was as follows: HbA1c < 7, 52.2%; from 7% to 7.9, 29.0%; from 8% to 8.9, 9.9%; and ≥9.0, 8.8% of patients [19].

Knowledge on the effectiveness of glucose-lowering therapy (GLT) in the Russian population has mainly been obtained from clinical trials; there are a lack of population studies in different regions and age ranges. In ageing, the size of the DM2 problem is rising. Therefore, in connection with changing approaches to the treatment of diabetes, permanent monitoring is relevant.

The aim of this study was to analyze the profile of GLT in persons with DM2 aged 55–84 years in a Russian population sample (Novosibirsk).

Early findings on drug therapy for atrial fibrillation, antihypertensive and lipid-lowering therapy in the studied population have previously been reported [20,21,22]. The present paper continues a systematic series of works on the pharmacotherapy of cardiometabolic diseases in the modern Russian population, using a population-based urban sample of older age persons in Novosibirsk.

## 2. Material and Methods

### 2.1. Participants

The study was performed on the material of a population cohort examined in Novosibirsk (the Russian arm of the multicenter project “Determinants of cardiovascular diseases in Central and Eastern Europe: cohort study”, the HAPIEE Project) [23]. A random sample of men and women aged 45–69 was drawn from residents of two districts typical of Novosibirsk in terms of infrastructure, demographic indicators and the level of population migration. The sample was formed on the basis of electoral lists using a table of random numbers and stratified by 5-year age groups; the design and protocol of the project have been published previously [23]. The design of the present work is a cross-sectional study. At baseline 9360 people were examined in 2003/05 (98% Caucasians, 61% response), the cohort was re-examined twice in 2006/08 and 2015/18. The present study focused on a sample of the third wave (*n* = 3898, age 55–84, response 60.1%). The analysis included 3896 people with the full required data set. The study was approved by the Ethics Committee of Research Institute of Internal and Preventive Medicine—Branch of the Institute of Cytology and Genetics, SB RAS. All participants signed an informed consent.

### 2.2. Study Questionnaire

The details of protocol and methods of the study have been published previously [23]. In brief, the protocol included the epidemiological assessment of cardiovascular diseases (CVD) and their risk factors using standardized questionnaires (medical history of hypertension and diabetes and their treatment, history of CVD and other chronic diseases, smoking, alcohol consumption, socio-demographic characteristics) and objective measurements (anthropometry, blood pressure measurement, electrocardiography, lipid and blood glucose levels).

A person who smoked at least 1 cigarette a day was considered a smoker. Alcohol consumption was assessed using the Graduated Frequency Questionnaire (GFR) and 5 groups were distinguished according to the frequency of consumption: non-drinkers, less than 1 time per month, 1–3 times per month, 1–4 times per week, 5 or more times per week.

### 2.3. Objective Measurements

Blood pressure (BP) levels were measured three times using an Omron M-5 tonometer on the right arm in a sitting position after a 5 min rest, with 2 min intervals between measurements. The mean value of three measurements of the office BP was used in analysis. Hypertension (HT) was defined according to the ESC/ESH criteria, 2018 [24], at systolic (SBP) or diastolic (DBP) BP levels ≥140/90 mmHg and/or taking antihypertensive drugs within the last 2 weeks.

Waist–hip ratio (WHR) and body mass index (BMI) were calculated using the formula:

BMI (kg/m^2^) = body weight (kg)/height^2^ (m^2^) (1)

WHR (units) = waist circumference/hip circumference(2)

A 12 lead ECG was recorded on electrocardiograph Cardiax (IMEDLtd., Budapest, Hungary) and assessed using the Minnesota code (MC).

Blood samples were collected at fasting stage; levels of total cholesterol (TC), triglycerides (TG), high-density lipoprotein cholesterol (HDL-C), and glucose in blood serum were measured by the enzymatic method on a KoneLab 300i autoanalyzer (Thermo Fisher Scientific, Waltham, MA, USA). The level of low-density lipoprotein cholesterol (LDL-C) was calculated using the Friedewald formula. The conversion of fasting blood serum glucose into fasting plasma glucose (FPG) was performed according to the formula of the European Association for the Study of Diabetes, 2007 [25]:

FPG (mmol/L) = −0.137 + 1.047 × serum glucose concentration (mmol/L) (3)

DM2 was established by having a history of DM2 with treatment and/or a FPG level ≥7 mmol/L [26]. All individuals with DM2 history or first ever revealed DM2, were included in the analysis of hypoglycemic therapy.

Coronary heart disease (CHD) was defined by epidemiological criteria: a positive score of the Rose Angina questionnaire or ischemic ECG changes (MC classes 1, 4, and 5) [27,28], or a medical history of myocardial infarction (MI), acute coronary syndrome, or coronary revascularization (confirmed by hospitalization). A composite CVD category was defined in the presence of CHD, based on above specified criteria or a history of stroke/transient ischemic attack (confirmed by hospitalization information).

The regular intake of hypoglycemic drugs was evaluated as a daily intake for the last 12 months without taking into account the dosage of the drug substance. Medicinal products were coded according to the Anatomical Therapeutic Chemical Classification System (ATC) [29]. The following drugs were included in the analysis: insulins (code A10A), biguanides (code A10BA), sulfonylurea drugs (SU, code A10BB), alpha-glucosidase inhibitors (iAG, code A10BF), thiazolidinediones (glitazones) (TZD, code A10BG), inhibitors DPP-4 (iDPP-4, code A10BH), glucagon-like peptide-1 receptor agonists (aGLP1, code A10BJ), inhibitors of sodium glucose cotransporter type 2 (gliflozins) (iSGLT-2, code A10BK), glinides (meglitinides) (code A10BX), and other drugs. Coding was performed by three specialists (cardiologists and endocrinologists). Reproducibility was assessed in a 10% subgroup by a double-blind fashion, and the Kappa agreement coefficient was 0.84.

A total of 3896 people were examined. At stage 1, the analysis included 803 persons with DM2. Of these, 476 persons were taking GLT and were included in the analysis at stage 2. The proportion of people who reported specific GLT was more than half—322 people. Next, we analyzed the proportion of glycemic control among all individuals with DM2 (including newly diagnosed) and among those taking GLT.

### 2.4. Statistical Analysis

Statistical analysis was carried out using the SPSS package v.13.0. Data are presented as means and standard deviation, M (SD), or as proportions, *n* (%). The frequency of the trait in the groups was compared using the χ^2^-Pearson test and the non-parametric Mantel–Hansel and Cochrane tests; ANOVA (analysis of variance) was used for quantitative comparisons. The Mann–Whitney test was used for abnormal distribution. Hypothesis testing was performed at a 95% confidence level for two-tailed tests.

## 3. Results

The general characteristics of the study sample aged 55–84, are presented in Table 1.

In the studied sample, the average age of the respondents was 69.3 years (SD 6.89) and was similar in men and women. The distribution of the participants by 10-year groups was 32.1% for the age of 55–64 years, 40% for the age of 65–74, and somewhat less (27.2%) for the group 75 years of age and older. Women, compared with men, had higher BMI and blood lipid values, a higher frequency of HT and antihypertensive therapy, and a similar prevalence of DM2 with a higher frequency of taking GLT; more often women had a low level of education and the status of “single.” Compared with women, men had higher levels of BP, WHR and FPG, a higher prevalence of CHD and CVD, and smoking and alcohol consumption was more common.

The prevalence of DM2 in the population sample aged 55–84 years was 20.8%, and was similar in men and women (20.1% and 21.2%, respectively, *p* = 0.463). Among subjects with DM2, 59.3% received GLT, women more often than men (66.1% vs. 47.8%, respectively, *p* < 0.001), Table 2. About 32% of subjects with DM2, including newly diagnosed diabetes, did not receive GLT, and another 8.8% did not provide information on GLT.

**Figure 1:**
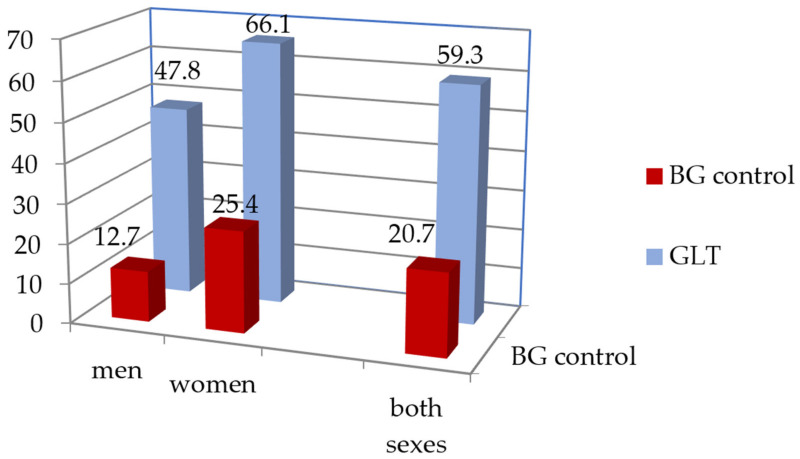
Frequency of glucose-lowering therapy (GLT) and blood glucose control (BG control) among people with type 2 diabetes, *n* = 803 (population sample, 55–84 years old).

FPG control < 7.0 mmol/L was achieved in every fifth participant with DM2 and in 35% of those taking GLT, Table 3, Figure 1. Participants who reported the name of a specific drug had control of glucose levels in about the same proportion (33%). Overall, women monitored their blood glucose levels more often than men.

In frequency of GLT use, biguanides ranked first place (75%), sulfonylurea derivatives in second place (35%), insulins in third place (12%), and DPP4 inhibitors in fourth place (5%). Combination GLT drugs were used by about one-third of individuals with DM2 (24% oral, another 6% in combination with insulin). One third of individuals with DM2 (including those newly diagnosed) did not receive GLT, which significantly affects the insufficient control of DM in the population.

## 4. Discussion

The prevalence of DM2 in the Novosibirsk population sample aged 55–84 years was 20.8%. Among persons with DM2, including newly diagnosed disease, 35% did not receive GLT. The target values of glycemia (FPG < 7.0%) were achieved in 20.7% of the group with DM2 and in 31.9% among those taking GLT. HbA1c levels were not assessed in this population study. Women effectively controlled glycemia more often than men.

In our study, in the entire DM2 group, we identified 35% of individuals with newly diagnosed diabetes who had not previously received GLT, which potentially contributes to a strong prediction of future CVD. The worsened prognosis is supported by the findings of a negative effect of prior hyperglycemia on coronary circulation, by an increased likelihood of having more severe and extensive CHD, and by the poorer profile of cardiometabolic risk factors in newly revealed DM2, compared with those with known diabetes [30,31].

It has also been reported that among patients with ST-elevation MI (STEMI) undergoing percutaneous coronary intervention (PCI), those with overt or newly diagnosed diabetes have a similar in-hospital and 3-year mortality rate, though mortality is lower in patients with pre-diabetes or no dysglycemia [32]. Patients with increased FPG or newly diagnosed diabetes following MI have an increased incidence of major adverse cardiac events (MACE) with the negative outcomes [33].

Plasma glucose measurement plays an important role in predicting adverse events, especially in subjects with previously unknown DM2 [34]. Given the above, more attention should be paid to individuals with newly diagnosed DM who should be screened for complications, particularly among those with a history of CV events.

The frequency of GLT use in our sample was expectedly lower compared with the EUROASPIRE-V study, which analyzed a sample of patients after a coronary event [15]. In the EUROASPIRE I–V study, 29% of all patients reported having DM2; of them, GLT was taken as follows: insulin, 32%; oral GLT, 74%; and 16% of the examined patients did not assess their blood glucose levels after discharge [15].

Our results were closer to the findings of a retrospective analysis of medical and pharmacological data on GLT use in the United States (more than 1.6 million, age 18–75+) [35], where the proportion of patients with diabetes who did not take GLT in the period 2006–2013 ranged from 25.7 to 24.1%.

The level of glycemic control among those receiving therapy in our sample was 35% (<7.0 mmol/L) and was approximately two times lower than the achieved control by the target HbA1c < 7.0% in the general EUROASPIRE-V cohort (54%) [15], and 1.5 times lower than in the Russian portion of the EUROASPIRE-V cohort (47%) [36]. At the same time, in the NHANES study, 2009–2014 [37], the results were close to our data. In particular, in the NHANES study, 2009–2014, the prevalence of intensive glycemic control was studied, taking into account the factors contributing to the achievement of the target level of HbA1C < 7.0%, such as duration of diabetes, smoking, comorbidities, disability, depression and taking the definite drugs, as well as socio-demographic factors. After adjusting, it was found that in the adjusted model, the frequency of intensive control was 23.5%, 32.5%, and 35.6%, for persons aged 50–64, 65–74, and 75+ years, respectively, with no significant difference by sex [37]. Lipska KJ et al., 2017, similarly, showed that less than half of the youngest patients (48.0%), but more than 60% of the oldest patients (61.6%) achieved the level of HbA1c < 7.0% [37]. Thus, older people have been shown to be treated more aggressively than young people to achieve HbA1c < 7.0% despite the presence of comorbidities and other factors.

In the Tromso study, which included 27,281 women and men aged 40–84 years, there was a linear increase in the prevalence of diabetes from 1994 to 2016. The overall prevalence of diabetes, including HbA1c ≥ 6.5%, increased from 3.2% to 5.9% in women and from 3.7% to 7.9% in men. According to the latest survey, the treatment goal (HbA1c ≤7.0% or <7.5%) was achieved in 43.8% of women and 38.5% of men using antidiabetic drugs, compared with 83.6% and 76.1% of women and men, respectively, who did not take antidiabetic drugs [38]. The authors found that target achievement was lower among patients using antidiabetics compared with non-users, which could be explained by less severe disease among non-users (i.e., diet-regulated diabetes).

According to the Russian DM Register, in 2017, the number of people who reached the level of HbA1c < 7.0% was 52.2% [20], which was higher, compared with our study, according to the other criterion of FGP < 7 mmol/L.

In the profile of hypoglycemic therapy in the Novosibirsk sample, about 90% of individuals treated for DM2 took oral agents, and 12% received insulin. The proportion of oral therapy in our study was higher, and insulin therapy two times lower, than in the general EUROASPIRE-V cohort where insulin therapy and oral GLT were 32% and 74%, respectively [15]. In the Russian sample of EUROASPIRE-V, the frequency of insulin therapy was close to ours, at 14.9%, and the proportion of oral therapy was lower, at 72.4% [36].

In our study, two-thirds of patients with DM2 received monotherapy, 30% took combined therapy, including near 24% who received oral drugs combination. These figures are close to the data from the Federal Russian Register, which showed that in the structure of DM2 therapy in the RF in 2017, the prescription of oral GLT prevailed (75.2% of patients), mainly in the form of monotherapy (46.8% of patients); 25.6% of patients received a combination of two drugs, and 2.8% a combination of three drugs. The number of patients with DM2 on insulin therapy in 2017 was 18.6%, of which 10.8% received insulin therapy combined with oral GLT, and 7.8% were on insulin monotherapy [19]. Among the oral agents in our study, metformin was predominantly used (75.2%), SU derivatives were in second place (35.4%), about 5% of people with DM2 took iDPP-4. TZD group, aGLP-1 and iSGLT-2 were not taken by the participants of the examined sample. Similar data with a slightly lower proportion of metformin use were shown by the Russian DM Register, where the most commonly prescribed drugs in monotherapy were metformin, 57.3%, and SU, 41.2%; in third place by prescription in monotherapy was iDPP-4, 1.0%. The remaining classes of glucose-lowering medications accounted for less than 1% of monotherapy: glinides, 0.5%; iSGLT-2, 0.1%; TZD group, aGLP-1 and iAG—less than 0.01%. The most frequent combinations of two glucose-lowering medications were metformin and SU (92.58%), and metformin and iDPP-4 (5.63%) [19].

In DIGAMI 2, metformin was not associated with lower CVD mortality, but it conferred a reduced risk of non-fatal MI or stroke in the short-term follow-up [39], and lower mortality rates and risk of death from neoplasms in the long-term period [40]. Metformin is considered cardioprotective, since treatment with this agent is associated with a lower risk of mortality (compared with sulfonylurea or insulin therapy) in patients with diabetes and heart failure or MI, and with a decreased risk of non-fatal MI or stroke in patients with diabetes and MI [39]. For example, although some studies found no increased risk of adverse outcomes in patients receiving sulfonylurea before an index event [41], other studies found that patients with diabetes and MI on sulfonylurea, at the time of admission for a CV event had higher CV risk compared with those receiving metformin [42]. According to a large-scale CVD-REAL study (300,000 patients with DM2 from national registers), in clinical practice in Europe and the United States in the structure of GLT for the period of 2015–2017, metformin was prescribed in 78.7%, SU derivatives in 38.7%, iDPP-4 in 33.3%, TZD in 8.9%, aGLP-1 in 20.3%, and insulin in 29.3%, of patients [43].

Lipska KJ et al., 2017, reported a retrospective study based on the U.S. Pharmacological Service, which analyzed the data for 1,657,610 individuals with DM2 (age 18–75+) from 2006 to 2013. During the study period, the use of metformin (from 47.6 to 53.5%), iDDP-4 (from 0.5 to 14.9%), aGLP-1 (from 3.3 to 5.0%) and insulin (from 17.1 to 23.0%) increased, but the proportion of SU (from 38.8 to 30.8%) and TZD (from 28.5 to 5.6%) (all *p* < 0.001) decreased. Increased insulin use was caused primarily by contribution of basal insulin analogs (10.9 to 19.3%; *p* < 0.001) and rapid-acting insulin analogs (6.7 to 11.6%; *p* < 0.001) while the use of human insulin products actually decreased (from 11.6% to 5.6%; *p* < 0.001). The proportion of diabetic patients who did not intake any GLT, decreased slightly (from 25.7% to 24.1%; *p* < 0.001). Considering the complexity of treatment, the use of oral monotherapy increased slightly (from 24.3 to 26.4%) and the use of multiple (two or more) oral agents decreased (from 33.0 to 26.5%), while the use of insulin alone and in combination with oral agents increased (from 6.0 to 8.5%, and from 11.1 to 14.6%, respectively; all values *p* < 0.001) [35].

Similar trends were observed in the Russian Federation, in 2013–2017. The prescription of metformin increased to 68.3% and insulin to 19.8%, and the share of SU decreased to 53.6% [19].

While alogliptin and lixenatide have shown safety in the EXAMINE study in the earlier phase after ACS, empagliflozin (EMPA-REG [44], liraglutide (LEADER [45]), and semaglutide (SUSTAIN-6 [46]) may offer an opportunity for effective secondary prevention of cardiovascular disease. In our region, the percentage of people receiving this therapy is extremely small, which yields a poor level of secondary prevention of CVD in patients with type 2 diabetes.

A recent analysis of patients in the U.S.A. showed no improvement in overall glycemic control and noted an increase in the proportion of patients with HbA1c ≥ 9.0% (from 9.9 to 12.2%; *p* < 0.001) between 2006 and 2013, despite the increased use of newer and more expensive glucose-lowering drugs among these patients [35]. These data, combined with our present results, highlight the urgent need to re-evaluate existing therapies for patients with DM2 in order to improve glycemic control.

In the group with effective glucose control, the frequency of metformin use as expected, was higher compared with the group with ineffective control. We found differences neither by the frequency of combined therapy nor by the average number of drugs depending on the effectiveness of glycemic control, in our sample.

## 5. Study Limitations

The present study had a number of limitations. In a population-based screening, we were not able to assess the level of HbA1c, and the level of blood glucose was measured at one visit, which may have affected the identification of DM2. However, this limitation was minimized by the standardized blood sampling procedure (8 h of fasting, the same personnel and storage protocol) and the performance of analyses according to a unified protocol on one autoanalyzer KoneLab 300i device (Thermo Fisher Scientific Inc., Waltham, MA, USA) using the same Thermo Fisher kits in a certified IIPM—Branch of IC&G SB RAS laboratory.

The antihyperglycemic profile was analyzed on the basis of self-assessment, which may have been a source of inaccuracy. However, about 70% of those receiving GLT named specific drugs; ATC coding was performed by three certified specialists (cardiologists and an endocrinologist); and in the 10% group, reproducibility was controlled with a double-blind approach (coefficient of agreement 0.84); this made it possible to eliminate significant errors in the results. Additionally, the present analysis did not include data on other drugs or comorbidities. The focus of the paper was SLT in a population-based sample of DM2, while the interaction between mentioned conditions and profile of DM2 treatment and glucose control, requires a specific analysis, and is not in the scope of current paper. Furthermore, non-inclusion of other diseases and drugs for the present analysis is unlikely to affect the estimates of coverage by GLT, drug profile and frequency of glucose control among persons with DM2 in a studied population.

Another potential limitation was that we were not able to take into account the regimen and dosage of drugs in a population epidemiological study, but this did not affect the assessment of the frequency of use and the profile of GLT, or the revealed fact of insufficient glycemic control in general. In addition, the applied epidemiological approach provided comparability with a number of population studies, including long-term studies for dynamic evaluation.

The results of the analysis are limited to the Novosibirsk sample and cannot be extrapolated to other Russian regions. At the same time, a typical urban population was studied, which had a country-specific epidemiological profile and medical care practices, as well as mortality rates close to the average Russian mortality rates, and the results allow us to state the insufficiency of GLT on the example of a certain Russian population.

The analysis was carried out in a sample of predominantly elderly people, which limits the generalization of the results, but given the highest incidence of DM2 in older age, the results informatively reflect the profile of GLT in a more susceptible part of the population.

Altogether, this study has several strengths. In general, GLT in the Russian Federation has been investigated by the Federal Register of Diabetes [19]. Data from 2016–2017 on key cardiometabolic factors in the secondary prevention of CVD, including DM2, have recently been discussed on the basis of the Russian sample of EUROASPIRE-V for patients after a coronary event [31]. As an advantage of the present study, we continued monitoring in the Siberian region and provided new knowledge on the assessment of the GLT profile and DM2 control in a non-selective Russian population. The analysis revealed a significant prevalence of undiagnosed DM2 and insufficient glycemic control. In addition, these findings were based on a large sample, for the first time establishing the magnitude of a lack in glucose control at a population level in Russia. In the management of DM2, the proportion of new glucose-lowering medications was shown to be small, and HbA1c monitoring was insufficient for appropriate glycemic control, which has a public health implication to define directions and strengthen efforts for diabetes control.

## 6. Conclusions

In a population sample of men and women 55–84 years old, examined in a typical Russian city in 2015–2018, the frequency of DM2 was about 21%. Glycemic control was achieved in every fifth participant with DM2 (fasting plasma glucose < 7.0 mmol/L) and in every third participant receiving GLT. Overall, women monitored their blood glucose levels better than men. In the GLT profile in terms of frequency of use, biguanides ranked first place, sulfonylurea derivatives ranked second place, insulins ranked third place, and iDPP-4 ranked fourth place. Combined GLT was used by about one third of individuals with DM2 (24%—oral, and another 6% in combination with insulin). One third of persons with DM2 (including those newly diagnosed) did not receive GLT, which significantly affects the insufficient control of diabetes mellitus in the population.

## Figures and Tables

**Table 1:** General characteristics of the studied population sample (men and women 55–84 years old, Novosibirsk, *n* = 3896).

Risk Factors	General Sample	Men	Women	*p* *
		Mean (SD)/*n* (%)		
Examined, *n*	3896	1499 (38.42)	2397 (61.58)	
Age, years	69.29 (6.89)	69.04 (6.95)	69.46 (6.85)	*0.061*
SBP, mmHg	145.72 (21.31)	146.88 (20.64)	145.0 (21.69)	***0.007***
DBP, mmHg	83.63 (11.37)	85.79 (11.82)	82.28 (10.87)	***<0.001***
Heart rate, b/min	71.75 (11.41)	71.34 (12.16)	72.01 (10.91)	*0.084*
BMI, kg/m^2^	29.47 (5.49)	27.76 (4.59)	30.55 (5.73)	***<0.001***
WHR, unit	0.90 (0.08)	0.95 (0.07)	0.87 (0.07)	***<0.001***
TC, mmol/L	5.46 (1.19)	5.17 (1.14)	5.65 (1.19)	***<0.001***
LDL-C, mmol/L	3.46 (1.06)	3.28 (0.99)	3.58 (1.08)	***<0.001***
HDLC, mmol/L	1.32 (0.39)	1.24 (0.38)	1.38 (0.38)	***<0.001***
TG, mmol/L	1.49 (0.92)	1.44 (0.89)	1.52 (0.94)	***<0.005***
FPG, mmol/L	6.34 (1.81)	6.41 (1.83)	6.29 (1.8)	***0.041***
HT, *n* (%)	3137 (80.9)	1162 (78.0)	1975 (82.6)	***<0.001***
Treatment of HT (among subjects with HT), *n* (%)	2399 (77.4)	723 (62.8)	1676 (86.1)	***<0.001***
DM2, *n* (%)	803 (20.8)	299 (20.1)	504 (21.2)	*0.463*
Treatment of DM2 (among subjects with DM2), *n*%	476 (59.3)	143 (47.8)	333 (66.1)	***<0.001***
CHD, *n* (%)	573 (14.9)	261 (17.5)	312 (13.2)	***<0.001***
CVD, *n* (%)	769 (19.9)	337 (22.6)	432 (18.2)	***0.001***
Menopause, *n* (%)	(-)	(-)	1924 (81.5)	*(-)*
Smoking, *n* (%)				***<0.001***
Smokers	714 (18.6)	572 (38.5)	142 (6.0)	
Former smokers	515 (13.4)	410 (27.6)	105 (4.4)	
Non-smokers	2619 (68.1)	504 (33.9)	2115 (89.5)	
Frequency of alcohol intake, *n* (%)				***<0.001***
2–4 times/week	47 (1.2)	40 (2.7)	7 (0.3)	
Once a week	413 (10.7)	326 (21.9)	87 (3.7)	
1–3 times/month	835 (21.7)	444 (29.9)	391 (16.6)	
Less than once a month	1609 (41.8)	413 (27.8)	1196 (50.6)	
Non-drinkers	944 (24.5)	263 (17.7)	681 (28.8)	
Education, *n* (%)				
Primary	246 (6.3)	86 (5.7)	160 (6.7)	***<0.001***
Professional	1063 (27.3)	335 (22.3)	728 (30.4)	
Secondary	1252 (32.1)	478 (31.9)	774 (32.3)	
University	1335 (34.3)	600 (40.0)	735 (30.7)	
Marital status, *n* (%)				***<0.001***
Single	1535 (39.9)	230 (15.5)	1305 (55.1)	
Married	2319 (60.2)	1258 (84.5)	1061 (44.9)	

Note: * *p* comparison by sex, for categorical variables—non-parametric Mann–Whitney test, for quantitative variables—Pearson’s χ^2^ test. SBP/DBP—systolic/diastolic blood pressure, HR—heart rate, BMI—body mass index, WHR—ratio of waist circumference/hip circumference, FPG—fasting plasma glucose, TC—total cholesterol, TG—triglycerides, HDL-C—high-density lipoprotein cholesterol, LDL-C—low-density lipoprotein cholesterol, HT—hypertension, DM2—diabetes mellitus type 2, CHD—coronary heart disease, CVD—cardiovascular diseases.

**Table 2:** The frequency of use of GLT and blood glucose control in persons with DM2 (population sample, 55–84 years old, *n* = 3896).

Parameters	General Sample	Men	Women	*p _m-w_*
Examined, *n*	3896	1499	2397	
DM2, *n* (%)	803 (20.8)	299 (20.1)	504 (21.2)	*0.463*
GLT among persons with DM2 (total), *n* (%)	476 (59.3)	143 (47.8)	333 (66.1)	***<0.001***
Not receiving GLT, *n* (%)	256 (31.9)	112 (37.5)	144 (28.6)	
No data on receiving GLT, *n* (%)	71 (8.8)	44 (14.7)	27 (5.4)	
Proportion of GLT with specified drugs, *n* (%)	322 (67.6)	87 (60.8)	235 (70.6)	***0.037***
Proportion of undifferentiated GLT, *n* (%)	154 (32.4)	56 (39.2)	98 (29.4)	
Effective control of blood glucose in subjects with DM 2 (total), *n* (%) *n* = 803	166 (20.7)	38 (12.7)	128 (25.4)	***<0.001***
Effective control of blood glucose (in those receiving GLT), *n* (%) *n* = 476	166 (34.9)	38 (26.6)	128 (38.4)	***0.013***
Effective control of blood glucose (in those who specified GLT drug, *n* (%) *n* = 322	102 (33.2)	21 (24.1)	81 (34.5)	*0.184*
Effective blood glucose control (in those with undifferentiated GLT), *n* (%) *n* = 154	64 (42.2)	17 (30.4)	47 (48.0)	*0.068*

Note: —*p* comparison by sex, Pearson’s χ^2^ test. GLT—glucose-lowering therapy, DM2—diabetes mellitus type 2.

**Table 3:** The profile of drug classes of GLT in persons with DM2 (population sample, 55–84 years old).

Classes of GLT in Persons Who Specified Medicinal Products, *n* = 322	Total	Men	Women	*p _m-w_*
GLT, *n*	322	87	235	
Insulins, total, *n* (%)	38 (11.8)	12 (16.1)	24 (10.2)	*0.146*
Biguanides, *n* (%)	242 (75.2)	60 (69.0)	182 (77.4)	*0.118*
Sulfonylureas, *n* (%)	114 (35.4)	35 (40.2)	79 (33.6)	*0.271*
Heterocyclic sulfonamides, *n* (%)	0 (0.0)	0 (0.0)	0 (0.0)	*--*
Alpha-glucosidase inhibitors, *n* (%)	0 (0.0)	0 (0.0)	0 (0.0)	*--*
Thiazolidinediones, *n* (%)	0 (0.0)	0 (0.0)	0 (0.0)	*--*
iDPP-4, *n* (%)	15 (4.7)	9 (10.3)	6 (2.6)	***0.003***
aGLP1, *n* (%)	0 (0.0)	0 (0.0)	0 (0.0)	*--*
iSGLT-2, *n* (%)	0 (0.0)	0 (0.0)	0 (0.0)	*--*
Combined oral drugs, *n* (%)	76 (23.6)	26 (28.9)	50 (21.8)	***0.042***
Combined GLT, total, *n* (%)	95 (29.5)	35 (40.2)	60 (25.5)	***0.010***

Note: —*p* comparison by sex, Pearson’s χ^2^ test. GLT—glucose-lowering therapy, DM2—diabetes mellitus type 2, iDPP-4—dipeptidyl peptidase-4 inhibitors, aGLP1—analogues of glucagon-like peptide-1 receptors, iSGLT-2—inhibitors of sodium-glucose cotransporter type 2.

## Data Availability

The data presented in this study are available in tabulated form on request. The data are not publicly available due to ethical restrictions and project regulations.
